# Theoretical analyses and experimental validation of the effects caused by the fluorinated substituent modification of DNA

**DOI:** 10.1038/s41598-020-57899-7

**Published:** 2020-01-24

**Authors:** Jun Koseki, Masamitsu Konno, Ayumu Asai, Naohiro Horie, Kenta Tsunekuni, Koichi Kawamoto, Satoshi Obika, Yuichiro Doki, Masaki Mori, Hideshi Ishii

**Affiliations:** 1 Department of Medical Data Science, Graduate School of Medicine, Osaka University, Osaka, 565-0871 Japan; 2 Department of Gastroenterological Surgery, Graduate School of Medicine, Osaka University, Osaka, 565-0871 Japan; 3 Bioorganic Chemistry, Graduate School of Pharmaceutical Sciences, Osaka University, Osaka, 565-0871 Japan; 4 Translational Research Laboratory, Taiho Pharmaceutical Co., Ltd, Tokushima, 771-0194 Japan; 5 Department of Surgery and Science, Graduate School of Medical Sciences, Kyushu University, Fukuoka, 812-8582 Japan

**Keywords:** Computational biophysics, Computational science

## Abstract

Halogen-modified nucleic acid molecules, such as trifluorothymidine (FTD) and 5-fluorouracil, are widely used in medical science and clinical site. These compounds have a very similar nucleobase structure. It is reported that both of these compounds could be incorporated into DNA. The incorporation of FTD produces highly anti-tumor effect. However, it is not known whether to occur a significant effect by the incorporation of 5-fluorouracil. Nobody knows why such a difference will occur. To understand the reason why there is large differences between trifluorothymidine and 5-fluorouracil, we have performed the molecular dynamics simulations and molecular orbital calculations. Although the active interaction energy between Halogen-modified nucleic acids or and complementary adenine was increased, in only FTD incorporated DNA, more strongly dispersion force interactions with an adjacent base were detected in many thermodynamic DNA conformations. As the results, the conformational changes occur even if it is in internal body temperature. Then the break of hydrogen bonding between FTD and complementary adenine base occur more frequently. The double helix structural destabilization of DNA with FTD is resulted from autoagglutination caused by the bonding via halogen orbitals such as halogen bonding and the general van der Waals interactions such as CH–${\rm{\pi }}$, lone pair (LP)–${\rm{\pi }}$, and ${\rm{\pi }}$–${\rm{\pi }}$ interactions. Therefore, it is strongly speculated that such structural changes caused by trifluoromethyl group is important for the anti-tumor effect of FTD alone.

## Introduction

Some types of nucleic acid modifications such as methylation and acetylation are known to directly affect the functions of DNA and RNA. These modifications play a critical role in DNA manipulation and can considerably affect DNA transcription and replication^1–4^. In addition, artificially modified nucleic acid molecules with halogen atoms, such as 5-fluorouracil (5FU) and trifluorothymidine (FTD), have been used in medical science and for clinical therapies^5–9^. The 5FU acts as an antitumor drug by inhibiting the formation of ribosomal RNA by its incorporation into RNA replacing uracil, or inhibiting DNA synthesis by preventing thymidylic acid synthesis^10–12^. Recent studies have shown that 5FU also gets incorporated into DNA, however, the association between this incorporation and the antitumor effects remains unknown. Conversely, FTD, which is incorporated with DNA, possesses a highly effective antitumor potency^13–15^ although its structure is similar to that of thymidine. The combination of FTD and tipiracil hydrochloride (FTD/TPI) has been approved by some organizations, such as the FDA (Food and Drug Administration of the United States), as a treatment for colorectal cancer^16^. The reason why FTD has antitumor effect is not known yet, although many studies about it have been reported^17–22^. It is said today that the reason is not to form a covalent bond with thymidylate synthase^17^ but to lead to DNA dysfunction by the incorporation of FTD into DNA from some experimental results^18–22^. In this way, despite all the different pharmacological mechanisms, the structures of the base moieties of 5FU and FTD are very similar. Why is FTD showing antitumor effect by the incorporation into DNA and is not 5FU showing? How interactions with surrounding nucleic acids does these molecules have after incorporation into DNA? To our knowledge, the reasons for these differences remain unclear. Thus, in this study, we per formed molecular dynamics (MD) and ab initio molecular orbital (MO) calculations to qualitatively determine the role and effects of the fluorinated groups in 5FU and FTD. We firstly obtained interaction energies and distances between some atoms using the simple model base pairs, and then we compared the stabilization structure of DNA duplexes with each energy minimization. After that, we performed thermal fluctuation analyses of these DNA duplexes with MD calculations, followed by analysis of the interbase interactions. Finally, theoretically predicted results were verified.

## Results and Discussion

### Simple base pair models for dissociation energies analyses

**Figure 1:**
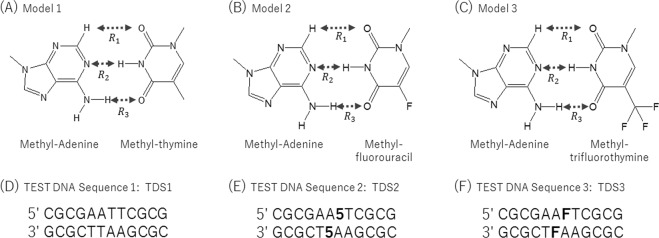
Simple base-pair models: methylated adenine and methylated thymine (**A**), methylated fluorouracil (**B**), and methylated trifluorothymine pair model (**C**). Test DNA Sequence 1 (**D**), 2 (**E**), and 3 (**F**).

We constructed simple base pair models, model 1, model 2, and model 3 (Fig. 1A–C), to analyze the difference in the hydrogen bonding intensity according to the presence or absence of the fluorine substituent and dissociation energies between thymidine–adenine, deoxyribose 5FU–adenine, and FTD–adenine pairs, respectively. These simple model structures were optimized by the second-order Møller–Plesset perturbation (MP2) using the 6-31++G** Gaussian basis set, followed by the dissociation energies. In this study, all *ab initio* molecular orbital calculations were performed with Gaussian 09^23^. Table 1 shows the distances, ${R}_{1}$, ${R}_{2}$, and ${R}_{3}$, in each stabilized structure, and the dissociation energies. For optimized structures of models 1–3, there is no striking difference in each distance. The stabilization energies were 16.3, 16.7, and 17.1 kcal/mol, respectively, which corresponded to the energies required to separate each molecular pair. These results demonstrate that the base pair was slightly stabilized by an increase in fluorine, and suggest that the attractive interaction is high owing to the electronic substituent effect caused by the fluorine group.

**Table 1:** Distances ${R}_{1}$, ${R}_{2}$, and ${R}_{3}$ in each optimized structure, and the dissociation energies.

		*R*_1_	*R*_2_	*R*_3_	Energy
		(Å)	(Å)	(Å)	(kcal/mol)
Model 1	−H…O−/−N…H−	2.72	1.80	1.95	16.3
	−N…N−/−N…O−	—	2.85	2.96	
Model 2	−H…O−/−N…H−	2.67	1.77	1.97	16.7
	−N…N−/−N…O−	—	2.83	2.98	
Model 3	−H…O−/−N…H−	2.68	1.77	1.97	17.1
	−N…N−/−N…O−	—	2.82	2.98	

### Double-stranded structures of 3 test DNA sequences for structural analyses

**Figure 2:**
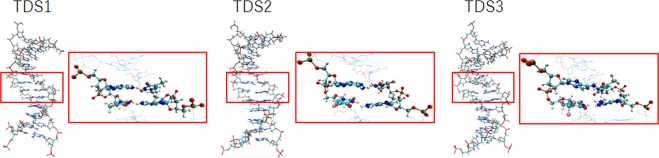
Minimization conformations of TDS1, TDS2, and TDS3, in the water phase. A magnified view of images for focused bases (ball and stick representation) are shown in each red frame.

We considered double-stranded structures of 3 test DNA sequences (TDS1, control sequence DNA; TDS2, DNA containing 5FU; and TDS3, DNA containing FTD) to determine the role of the fluorine group in the DNA (Fig. 1D–F). We adopted the short DNA sequence used by Markley *et al*.^24^ as a reference for these test DNA constructs. These DNA structures were constructed based on the B-type DNA, which is right-handed with about 10 base pairs per turn. For each the DNA structure in water molecules, energy minimizations were performed using the AMBER 12^25^ program package. The AMBER99^26^, general AMBER force field (GAFF)^27^ and TIP3P^28^ force fields for the complete DNA and water molecules were used respectively. Figure 2 shows the stabilization conformations for TDS1, TDS2, and TDS3, respectively. Disregarding thermal energies, we observed substantial differences between TDS1, TDS2, and TDS3, as shown in Fig. 2. A magnified view of images is shown in Fig. S1. In particular, the structure of TDS3 containing FTD became more distorted compared with TDS1, (the control sequence). Figure S2A shows the superposition of minimization structures with the tube representation for TDS1 (cyan) and TDS3 (red; the sphere representation implies the position of the fluorine atom). While the double-stranded structure was maintained in TDS1, the hydrogen bonding system between FTD and its complementary adenine broke in TDS3. In TDS2, we did not observe broken hydrogen bonding patterns, but a little distortion occurred in their double-stranded structure. By comparing with results of simple base pair models and test DNA sequences, the destabilization of the double-stranded DNA including FTD, was assumed to not change the hydrogen bonding intensity but the interactions between the trifluoromethyl group and surrounding bases. In previous report, W. H. Gmeiner *et al*.^29^ performed the energy minimization simulations for the double helix structure containing FTD. They reported that the trifluoromethyl group does not interfere with base pairing or base stacking, nor does it significantly affect helical dimensions. However, their calculation could not include the solvent effect. In general, it is known that, when the energy minimization of strongly charged molecules such as nucleic acids in gas phase, the hydrogen bonding and electrostatic interaction is overestimated. Therefore, in discussing the structure of the DNA including FTD, it is shown that consideration of solvent effect is indispensable.

**Figure 3:**
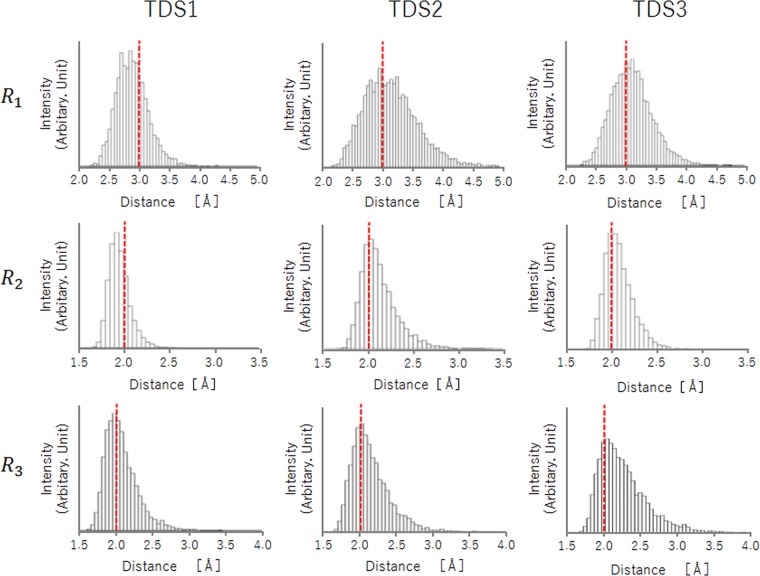
Distributions of the distance of ${R}_{1}$, ${R}_{2}$, and ${R}_{3}$ in TDS1, TDS2, and TDS3 in 310 K. The unit is in Å. The distance of 3.0 Å is an upper limiting length to form a weak hydrogen bonding. It is necessary to keep a distance of ≤2.0 Å for forming a strong hydrogen bond.

In addition, to elucidate the differences in thermodynamic behaviors in water phase, we performed MD simulations. Supplementary Movies S1–S3 present the thermodynamic behaviors of each TEST DNA. We focused on distances between the complementary base pairs to analyze the difference in these behaviors. Figure 3 shows the distributions of the distances of ${R}_{1}$, ${R}_{2}$, and ${R}_{3}$ in TDS1, TDS2, and TDS3, respectively. In terms of the ${R}_{1}$ distribution, the peak in TDS3 was longer than 3.0 Å, but in TDS1 it was shorter than 3.0 Å. In TDS2, the peak was around 3.0 Å, but the rate located at >3.0 Å was higher than those in other DNAs. The distance of 3.0 Å is the upper limit to form a weak hydrogen bonding^30^. We found that the ${R}_{2}$ distribution had peaks in TDS2 and TDS3 of around 2.0 Å, and longer than the peak in TDS1. In addition, the distribution of TDS3 was quite similar to that of TDS2. The ${R}_{3}$ distribution peaks were similar at around 2.0 Å. Although TDS1 and TDS2 exhibited analogous distributions, the probability of being >2.0 Å in TDS3 was much higher than that in the other test DNA sequences. In this case, it is necessary to maintain a distance ≤2.0 Å to form a strong hydrogen bonding. The ${R}_{1}$ distance in TDS2 was longer than that in TDS3. However, as weak hydrogen bonding could not be formed at a distance >3.0 Å, no marked differences existed in the thermal stability between TDS2 and TDS3. However, the rate of existence of ${R}_{3}$ at a distance <2.0 Å was higher in TDS2 than in TDS3. In addition, it was easier to take a distance >3.0 Å in TDS3 than in TDS2. Hence, it is more difficult for TDS3 to form a strong hydrogen bonding than for TDS2, and it takes more time to form a weak hydrogen bonding. Of note, TDS3 sometimes does not form hydrogen bonding. In other words, the hydrogen bonding between FTD and its complementary base in TDS3 was weaker than that in TDS2, and the breakage of hydrogen bonding occurs often. In TDS2, although the distance of ${R}_{1}$ which originally has a long interatomic distance distribution is broad, but the ${R}_{3}$ distribution is not significantly different from the distribution in TDS1. On the other hand, in the distance distributions of both ${R}_{2}$ and ${R}_{3}$ of TDS3, many are distributed beyond the distance region forming strong hydrogen bonding (2.0 Å) compare with TDS1. In other word, it means that the double helix structural stabilization of TDS3 is not maintained by forming hydrogen bonding while the stabilization of TDS2 is preserved.

**Figure 4:**
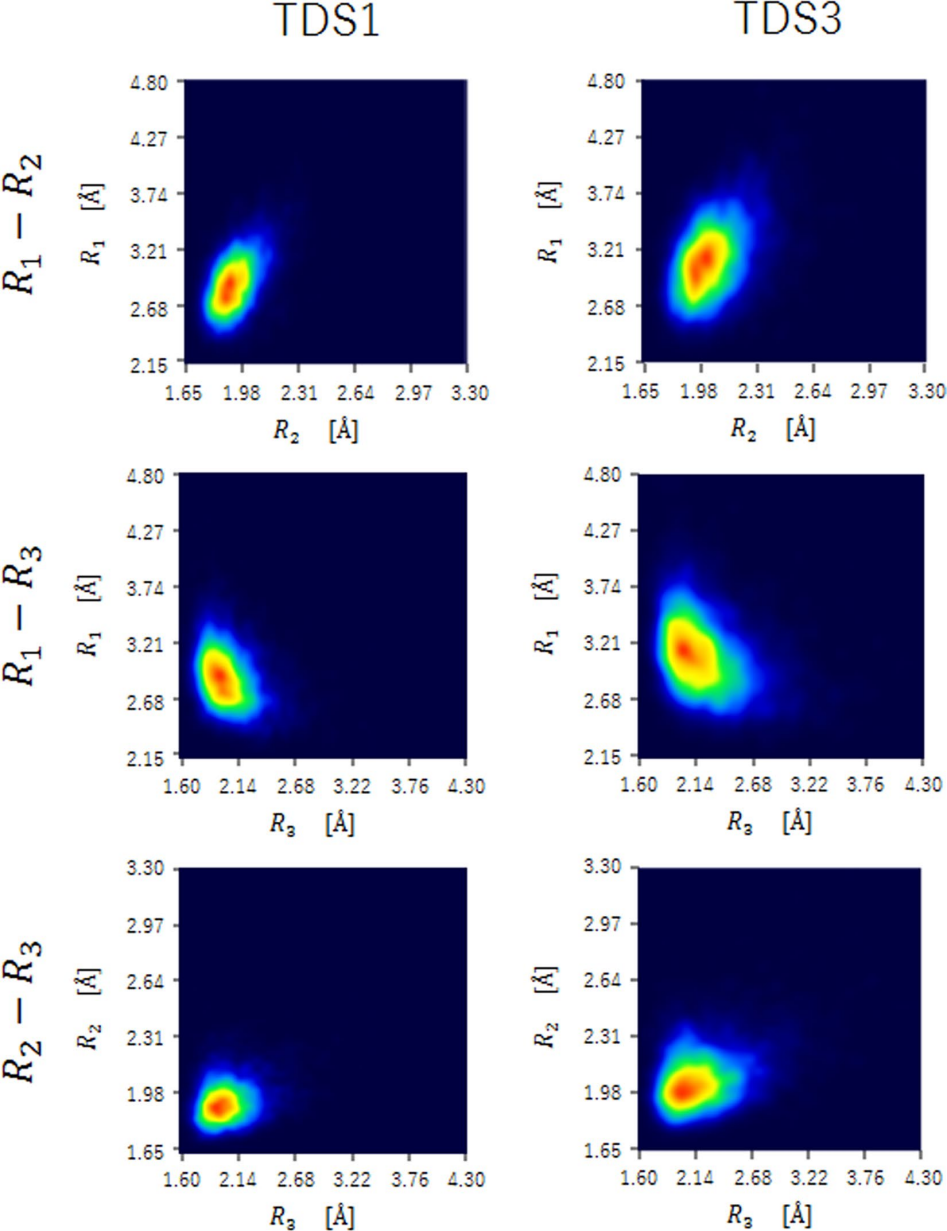
Thermodynamic two-dimensional distributions of the distance Å combination of ${R}_{1}$, ${R}_{2}$, and ${R}_{3}$ in TDS1 and TDS3.

Therefore, in order to understand hydrogen bonding instability in TDS3, molecular oscillation between FTD and complementary Adenine was focused on. The two-dimensional distribution of ${R}_{1}$-${R}_{2}$, ${R}_{1}$-${R}_{3}$, and ${R}_{2}$-${R}_{3}$ in TDS1, and TDS3 were shown in Fig. 4. As shown in ${R}_{1}$-${R}_{2}$ distributions, the ${R}_{1}$ distance stretched in step with the ${R}_{2}$ distance. The ${R}_{2}$-${R}_{3}$ distributions exhibited the same tendency as ${R}_{1}$-${R}_{2}$ distributions. Conversely, ${R}_{1}$-${R}_{3}$ distributions exhibited a different propensity than these distributions, and the ${R}_{1}$ distance stretched inversely to the ${R}_{3}$ distance. Notably, these tendencies could be observed in common. The difference between each DNA implies the spread of distributions. We observed widespread distributions in TDS3 compared with the distributions in TDS1. In TDS3, the correlation between ${R}_{2}$ and ${R}_{3}$ was comparatively larger than in TDS1 and the distance of ${R}_{2}$ expanded with the extension of the distance of ${R}_{3}$. As mentioned, the two-dimensional distance distributions in TDS3 exhibited an extensive presence compared with the distributions in TDS1 due to the existence of the interaction by the fluorine substituent. Typically, thermal vibrations of base pair are categorized into normal modes, such as buckle mode, propeller mode, opening mode, shear mode, stretch mode, and stagger mode^31,32^. In the thermal oscillation of the adenine–FTD pair in the MD trajectory of TDS3, many opening modes were observed (Supplementary Movies S4). This could be seen from the following behavior. As the ${R}_{1}$ distance increases in length, the ${R}_{3}$ distance shortens, and the ${R}_{1}$ distance decreases with an increase in the length of the ${R}_{3}$ distance. It might be that this opening mode induces hydrogen bonding fracture of TDS3. In fact, we could observation that water molecules are likely to enter into between two bases in molecular dynamics simulations.

### Interbase interaction analyses

**Figure 5:**
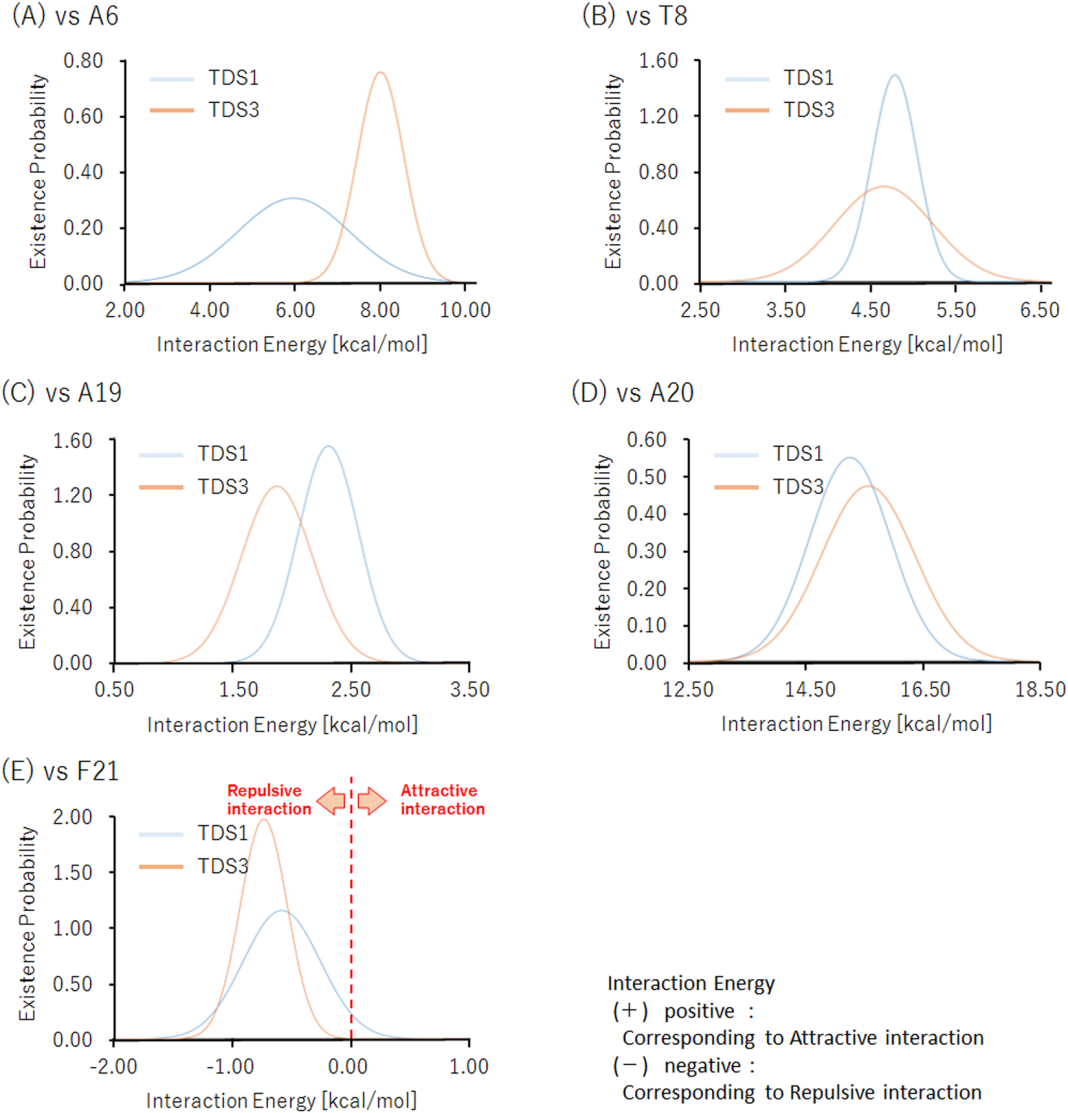
Gaussian approximated distributions of interaction energies between “T or F07” and “(**A**) A06, (**B**) T08, (**C**) A19, (**D**) A20, or (**E**) F21”. The positive and negative interactions are corresponding to attractive and repulsive interactions, respectively. The unit is in kcal/mol.

We performed interaction analyses between bases to analyze the factors causing a hydrogen bonding fracture in TDS3. Thus, we introduced the conformational clustering in eight bases, 5th-adenine (A05), 6th-adenine (A06), 7th-thymidine or FTD (T07 or F07), 8th-thymidine (T08), 19th-adenine (A19), 20th-adenine (A20), 21st-thymidine or FTD (T21 or F21), and 22nd-thymidine (T22) to elucidate the possible conformational distribution in MD simulation trajectories (5000 conformations) for TDS1 and TDS3. Eight bases (5A, 6A, “7T or F”, 8T, 19A, 20A “21T or F”, and 22T) were selected from the trajectories of MD simulations for TDS1 and TDS3, respectively, to analyze their interbase interaction energies. Thereafter, we proposed the partial base models comprising these eight bases. Only the hydrogen atoms were reoptimized with MP2/6-31 G* level. Then, the distribution of interaction energies was evaluated to estimate interaction energies between eight bases, each base energy, and any pair energy using each of the models at the Hartree-Fock (HF) and MP2/6-31 G* level. It is well known that HF method is unstable for the description of stacking interactions^33^ since the method could not restage the dispersion force interactions. However, we venture to estimate the energies with HF method in order to analyze using the difference of the characteristic of MP2. Figure S3 shows the superposition of 5000 conformations in each MD simulation. The variation areas of fluorine atoms in F07 and F21 were more extensive than that of hydrogen atoms in T07 and T21. Table S1A,B presents the interbase interaction energies for TDS1 and TDS3 using five representative conformations selected by conformational clustering. Figure S4A,B shows the structural clustering dendrogram for TDS1 and TDS3. Under these energies, the positive and negative values corresponded to attractive and repulsive interactions, respectively. In TDS1, the sign of interaction energies between T07 and T08 differed from that between HF and MP2. In TDS3, not only the F07-T08 interaction but also the F07-A06 interaction with MP2 were opposite in sign to the interactions with HF. The MP2 calculations can include the effect of dispersion interaction although HF cannot. In other words, it means that given that the estimated energy reflects the contribution of dispersion interaction, the signs of energy levels in HF and MP2 must be different. Figure 5A–E shows the Gaussian approximated distributions of the interaction energy with the MP2 level; we found apparent differences in each interaction energy between TDS1 and TDS3. The A06 interaction in TDS3 was strong and its range shrunk compared to the one in TDS1. The interaction energy peaks for T08 were similar in both TDS1 and TDS3. However, the distribution of TDS3 was more extensive than those in TDS1, implying that TDS3 had some conformations with interaction energies stronger than TDS1. In contrast, for A19 within the complementary strand, the distribution of TDS3 tended to locate in a lower energy region than that of TDS1. In A20, interaction energy distributions were similar to the model system; FTD within TDS3 had a strong interaction energy with the complementary base compared with the thymidine within TDS1. Finally, the interaction energy of F21 was basically negative in TDS3; however, TDS1 exhibited positive interaction energies. In other word, FTD attracts adjacent bases on the same chain strongly, although the interactions with complementary strand bases are weaker than in TDS1 or even repulsive. The DNA duplex instability of TDS3 is induced by these complex factors. In addition, we have also performed natural bond orbital (NBO) analyses^34–36^ to determine the difference in their interaction energy intensities. In the A06-F07 and F07–T08 interaction, we found some electronic interactions caused by the halogen bonding and hydrogen bonding via halogen besides the general van der Waals interactions such as CH–${\rm{\pi }}$, lone pair (LP)–${\rm{\pi }}$, and ${\rm{\pi }}$–${\rm{\pi }}$ interactions. The self-cohesive properties due to halogen bonding interactions and hydrogen bonding via halogen orbitals in TDS3 is a unique property not shared by TDS1, which we consider the essential factor causing double-stranded DNA destabilization. Although the conformational change occurs during thermal agitation, the trifluoromethyl group exhibits interactions by the bonding via halogen orbitals to A06 or T08. In fact, when the conformation estimated the minimum interaction energy between F07 and A06, we found the bonding interactions via halogen orbitals to T08.

Although the thermodynamic conformational sampling with molecular dynamics were carried out in the water phase, the above estimations of interaction energies were calculated in the gas phase environment. Therefore, in order to confirm the influence of the solvation effect on the interaction energy estimations, we also tried to estimate the interaction energies with the IEFPCM method for five representative conformations selected by conformational clustering. In a biological environment, it can be assumed that DNA exist in water phase, but individual bases in DNA are sandwiched between other bases having permittivity lower than that of water. Therefore, as a solvent, in addition to the case of water, we considered the case of benzene and DMSO, which have a lower permittivity than water. Figure S5 and Table S2 show the interbase interaction energies between T/F07 and surrounding bases with MP2/6-31 G* level. These results means that although the interaction energy values generally changes depending on the solvent, there is no qualitative difference in the distribution of interaction energy. Therefore, our results even for gas phase approximation in qualitative discussions at the previous paragraph are considered sufficiently reliable.

### Difference of thermodynamic parameter

As these results, it can be predicted that DNA containing FTD has a lower melting temperature (${T}_{m}$) of the double helix structure than normal type DNA and DNA containing 5FU. Therefore, we determined the thermodynamic parameters, such as ${T}_{m}$, $-\Delta H$, and $-\Delta S$, of a series of duplexes comprising TDS1, TDS2, and TDS3 by UV melting experiments, to experimentally verify the thermodynamic behavior predicted from the theoretical calculations. Table 2 shows the melting temperature (${T}_{m}$), change in enthalpy ($-\Delta H$), and entropy ($-\Delta S$) for each TDS. The values of ${T}_{m}$ for TDS1, TDS2, and TDS3 were 64.2, 58.9, and 56.8 °C, respectively. The ${T}_{m}$ value decreased by approximately 5 °C (or 7 °C, after 5FU (or FTD) incorporation into the DNA. Moreover, the values of enthalpy change and entropy change were altered according to the change in the ${T}_{m}$ value. In other word, DNA duplexes containing 5FU (TDS2) or FTD (TDS3) are unstable compared with the normal DNA (TDS1). In addition, TDS3 is slightly less stable than TDS2. As already mentioned, the interbase interactions substantially change because of the fluorine substituent incorporated into the DNA. We believe these thermodynamic experimental results are due to a change in the hydrogen bonding properties between the complementary strands. Therefore, it might be that the incorporation of FTD into the DNA causes a strong attraction in the nucleic acid chain that breaks the hydrogen bonding to the complementary base, resulting in the loss of thermal DNA stability. It is possible that this difference between the normal DNA and the one incorporating FTD changes the affinity of the DNA binding protein for the molecule, leading to transcription and replication inhibitions similar but stronger to those due to DNA methylation. These physical and biological behaviors may cause the unique pharmaceutical actions of FTD as shown in Fig. S6. Although it is interested in the association between DNA binding protein and FTD-included DNA, experimental verifications to investigate above behaviors are very difficult. Therefore, in order to understand these considerations in detail, we are now developing a novel method for predicting the binding mode between DNA recognition protein and any DNAs with arbitrary sequence.

**Table 2:** Thermodynamic analyses for TDS1, TDS2, and TDS3.

		TDS1	TDS2	TDS3
*T*_m_	(°C)	64.3	58.9	56.8
-*ΔH*	(kcal/mol)	50.7	38.1	31.9
-*ΔS*	(cal/mol/K)	130	94.4	76.2

### Antitumor drugs administration test

If the thermal instability of the DNA is related to the pharmacological mechanism, FTD should be effective not only for colorectal cancer but also for other various cancer types. Thus, we performed antitumor drug administration tests with a subcutaneous implantation model in nude mice using PANC-1 cells (a human pancreatic adenocarcinoma cell line). Figure S6 shows the results of the time course observation for the relative body weight rate (A) and for the relative tumor volume rate (B). We observed no difference in the relative body weight rate between control, 5FU (S-1), and FTD (trifluorothymidine/tipiracil, TAS-102) groups. As mentioned above, the pharmacological effect of 5FU is not caused by incorporation into DNA like FTD. Then although we could not do a simple comparison with, we found that marked differences in the relative tumor volume rate between these groups. Although we showed antitumor effects in both 5FU and FTD groups, the FTD group exhibited a higher effect than the 5FU group after 24 days. Table S3 presents these numerical data along with the results using gemcitabine (GEM), one of the antitumor drugs for pancreatic cancer; as shown in this table, the FTD group exhibited an antitumor effect potency equivalent to or higher than that in the GEM group. As expected, our results revealed that FTD suppresses the tumor volume increase without causing more weight loss. Since the thermal instability by the FTD incorporation into the DNA causes its antitumor effects, FTD should cause antitumor effects regardless of the cancer type.

## Conclusion

By using theoretical methods such as molecular dynamics and quantum chemistry, we could show the difference of intra-DNA interaction and the change of the 3D conformation by incorporation of nucleic acid having fluorine substituent into DNA. Then, the results that might clarify the pharmacological effect were found out. The incorporation of FTD into DNA destabilizes the conformation of double-helical DNA via halogen bonding and dispersion interactions, therefore, the FDT incorporated DNA will result in alterations in affinity to protein bindings. Thus, the calculation technology is a bona fide useful entity to raise the standard of re-searches in life science.

## Methods

### Energy minimization and Molecular dynamics simulation

DNA structures of TDS1, TDS2, and TDS3 based on the B-type DNA were constructed, to analyze the conformational change caused by each the fluorine substituent in TDS2 and TDS3, compared with TDS1. The B-type DNA is right-handed with about 10 base pairs per turn. For each the DNA structure in about 6200 water molecules, energy minimizations was performed using the AMBER 12 program package. The AMBER99, GAFF, and TIP3P force fields for the complete DNA and water molecules were used respectively. In particular, a general AMBER force field (GAFF) was used for 5FU and FTD. After the minimization calculations, the NVT MD simulations were performed at 310 K with the periodic boundary condition from minimized structures to obtain a sample for confirming each DNA structure. The time step and total simulation time were 0.2 fs and 10 ns (50,000,000 steps), respectively.

### *Ab initio* molecular orbital method

Simple base pair models, such as adenine–thymidine, adenine–fluorouracil, and adenine–FTD, were constructed to analyze the difference in the hydrogen bonding intensity according to the presence or absence of the fluorine substituent. In these models, a methyl group substituted each 1st-carbon in ribose. These simple model structures and simple mode bases were optimized by MP2 using the 6-31++G** Gaussian basis set, followed by the stabilization (dissociation) energies ($\Delta {E}_{Dissoc}$). In addition, separated each molecular structure was optimized. We estimated each dissociation energy with the energies of optimized structures ($\Delta {E}_{Dimer}$, $\Delta {E}_{Adenine}$, $\Delta {E}_{Thimidinelike}$) using the following equation.

$$\Delta {E}_{Dissoc}=(\Delta {E}_{Adenine}+\Delta {E}_{Thimidinelike})-\Delta {E}_{Dimer}$$

In the interbase interaction analyses, eight bases (5A, 6A, “7T or F”, 8T, 19A, 20A “21T or F”, and 22T) were selected from the trajectories of MD simulations for TDS1 and TDS3, respectively. Thereafter, we proposed the partial base models comprising these eight bases. Each 1st-carbon in ribose was the substituted hydrogen atom in these models. Only the hydrogen atoms were reoptimized. Interaction energies were estimated using above the equation for dissociation energy although in these interaction energies calculation the molecular structures were fixed. Then, the distribution of interaction energies was evaluated to estimate interaction energies between eight bases, each base energy, and any pair energy using each of the models at the MP2/6-31 G* level. In addition, we performed the interaction energies’ estimation with solvation effects using the IEFPCM method. For comprehensively analyzing the electronic interaction, NBO analyses were used. In this study, all ab initio molecular orbital calculations were performed with Gaussian 09.

### Thermal denaturation experiments for intended DNA

Thermodynamic data, such as ${T}_{m}$, $\Delta H$, and $\Delta S$, were estimated for TDSs with ultraviolet and visible spectrophotometry (SHIMADZU UV-1800 spectrometer) equipped with Tm analysis accessory quartz cuvettes with 0.1 cm optical path length. The samples were prepared in 10 mM sodium phosphate buffer (pH 7.2) containing 100 mM NaCl and 17–70 μM of each oligonucleotides at the final concentration, annealed by heating at 100 °C, followed by slow cooling to room temperature. The melting profile was measured at 260 nm at 5–90 °C at the scan rate of 0.5 °C/min. In addition, we used the two-point average method to determine the ${T}_{m}$ values, and the final values were determined by averaging three independent measurements that were accurate within 1 °C.

### Antitumor drugs administration test with subcutaneous implantation model mouse

This study was approved by the Animal Experiments Committee, Osaka University (approval number: 30-011-008). Our all experiments were performed in accordance with relevant guidelines and regulations. We purchased 6-week-old female Crlj:SHO-PrkdcscidHrhr mice from Charles River Laboratories Japan, and maintained them in a specific pathogen-free room. After 1 week, PANC-1 cells (1.0 × 107 cells/150 μL) were subcutaneously transplanted under the skin of their left inguinal part. Once the subcutaneous tumor volume had reached 50 mm^3^, we initiated anticancer drug administrations. Control mice got 10 mL/kg of D-PBS(–) injected into the tail vein once a week. GEM mice got 10 mL/kg of gemcitabine hydrochloride (10 mg/mL) injected into the tail vein once a week. 5FU (S-1) mice got tegafur, gimeracil, and oteracil at a weight ratio of 1:0.29:0.98; 10 mL/kg of this combination (S-1; 0.83 mg/mL) were administered orally once a day for a week. FTD (FTD/TPI, TAS-102) mice got trifluridine and tipiracil hydrochloride at a weight ratio of 1:0.47; 10 mL/kg of this combination (FTD/TPI; 15 mg/mL]) were administered orally once a day for 1week. We evaluated the subcutaneous tumor volume as follows using the following formulas: (greatest diameter) × (shortest diameter)^2^/2.

## Supplementary information

Supplementary Movie 1.

Supplementary Movie 2.

Supplementary Movie 3.

Supplementary Movie 4.

Supplementary Infomation.
