# Supplementary material for: Theoretical analyses and experimental validation of the effects caused by the fluorinated substituent modification of DNA
Source: Sci Rep. 2020 Jan 24;10:1138. doi: 10.1038/s41598-020-57899-7 (PMC6981298; doi:10.1038/s41598-020-57899-7)
Supplement: Supplementary file 5 — Supplementary Infomation. [file 41598_2020_57899_MOESM5_ESM.pdf]

Theoretical analyses and experimental validation of the effects caused by the fluorinated substituent modification of DNA

Jun Koseki[1], Masamitsu Konno[2], Ayumu Asai[1,2], Naohiro Horie[3], Kenta Tsunekuni[1,2,4], Koichi Kawamoto[2], Satoshi Obika[3], Yuichiro Doki[2], Masaki Mori[2,5], Hideshi Ishii[1,*]

[1] Department of Medical Data Science, Graduate School of Medicine, Osaka University, Osaka, 565-0871 Japan.

[2] Department of Gastroenterological Surgery, Graduate School of Medicine, Osaka University, Osaka, 565-0871 Japan.

[3] Bioorganic Chemistry, Graduate School of Pharmaceutical Sciences, Osaka University, Osaka, 565-0871 Japan.

[4] Translational Research Laboratory, Taiho Pharmaceutical Co., Ltd., Tokushima, 771-0194 Japan.

[5] Department of Surgery and Science, Graduate School of Medical Sciences, Kyushu University, Fukuoka, 812-8582, Japan

Supplementary Figure

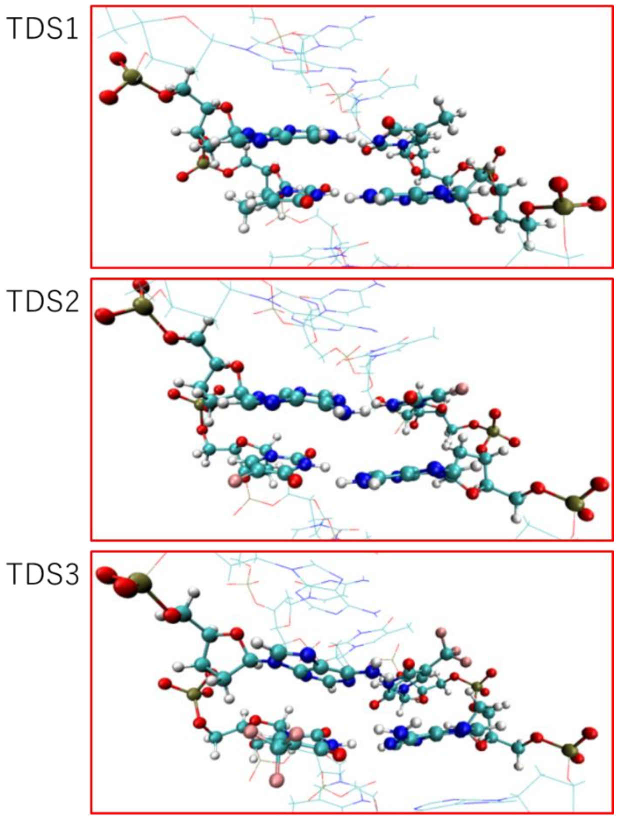

Supplementary Figure 1. A magnified view of images for focused bases (ball and stick representation) of minimization conformations of TDS1, TDS2, and TDS3, in the water phase.

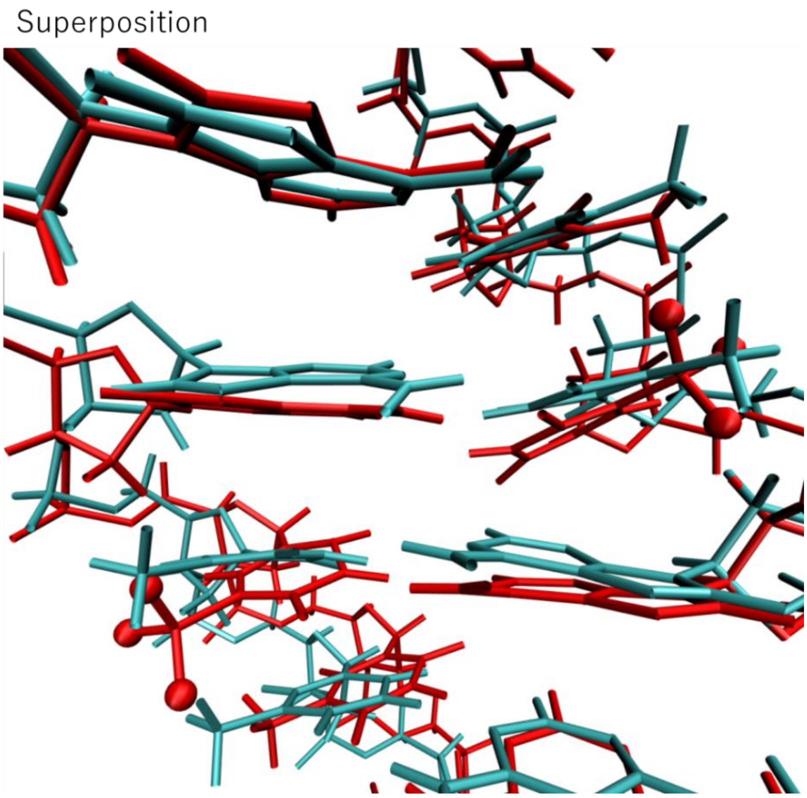

Supplementary Figure 2. Superposition of minimization structures for TDS1 (cyan tube representation) and TDS3 (red tube representation). In TDS3, the red sphere shows the position of the fluorine atom.

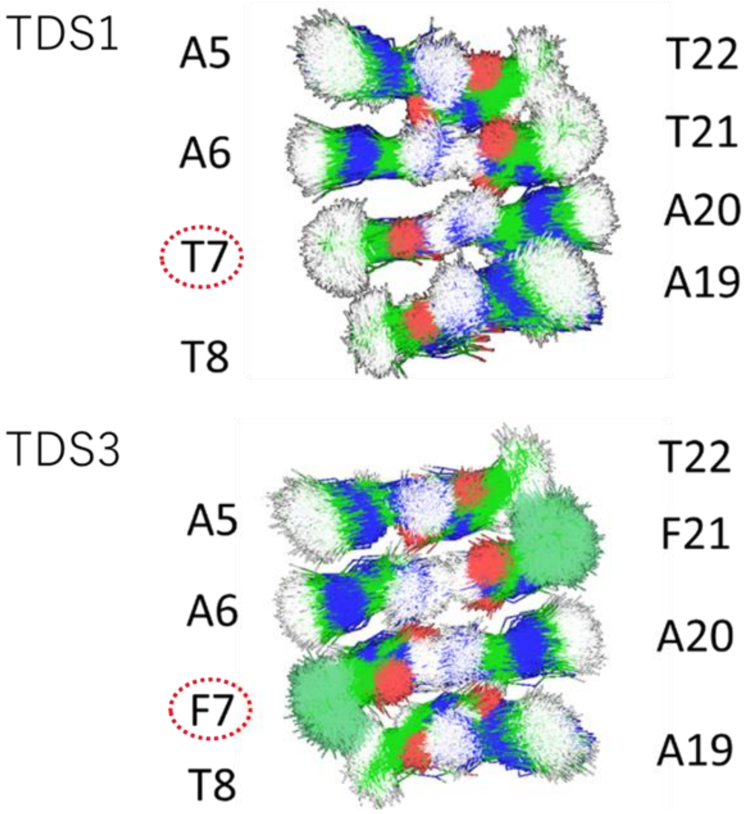

Supplementary Figure 3. The superposition of line representations of trajectory for TDS1 and TDS3.

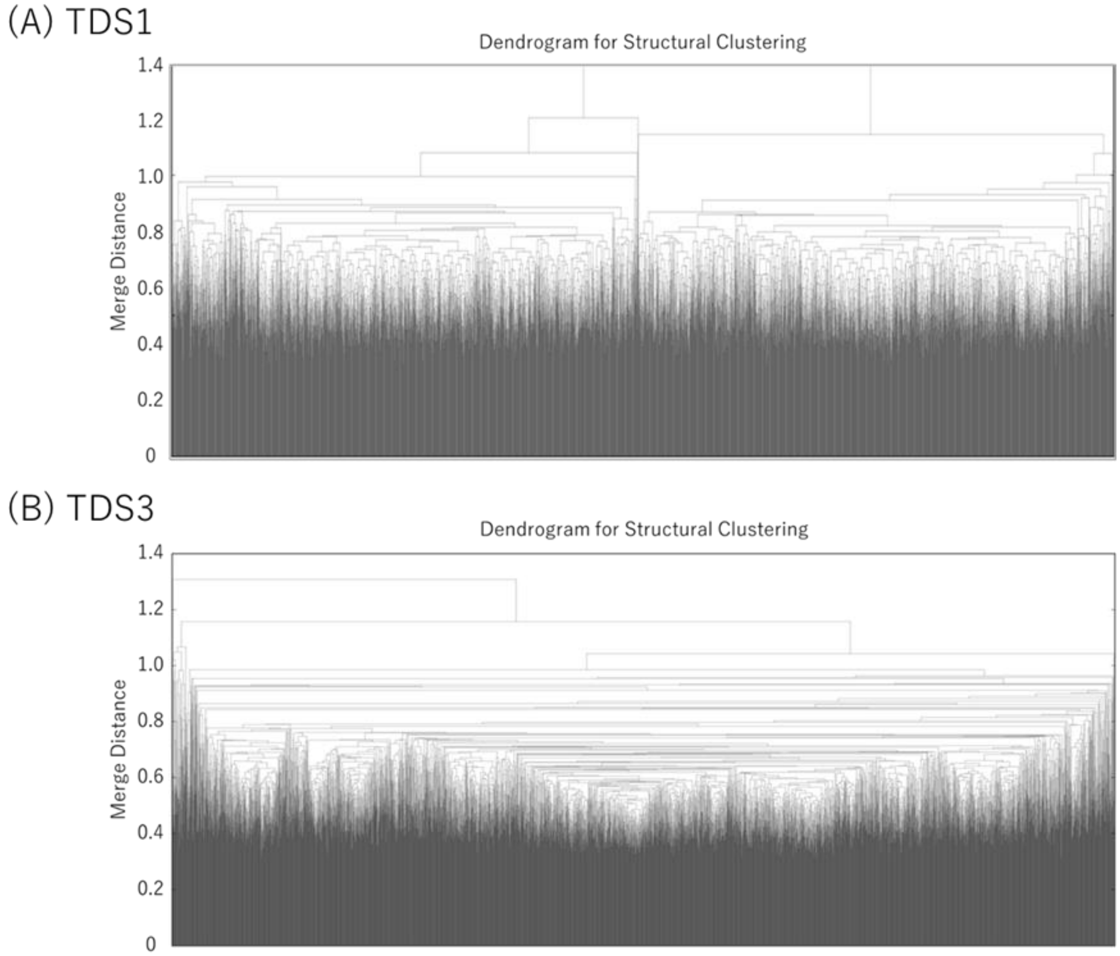

Supplementary Figure 4. Structural clustering dendrograms of eight bases (5th-adenine, 6th-adenine, 7th-thymidine or FTD, 8th-thymidine, 19th-adenine, 20th-adenine, 21st-thymidine or FTD, and 22nd-thymidine) using each 5000 conformation obtained from molecular dynamics simulations' trajectory for TDS1 and TDS3.

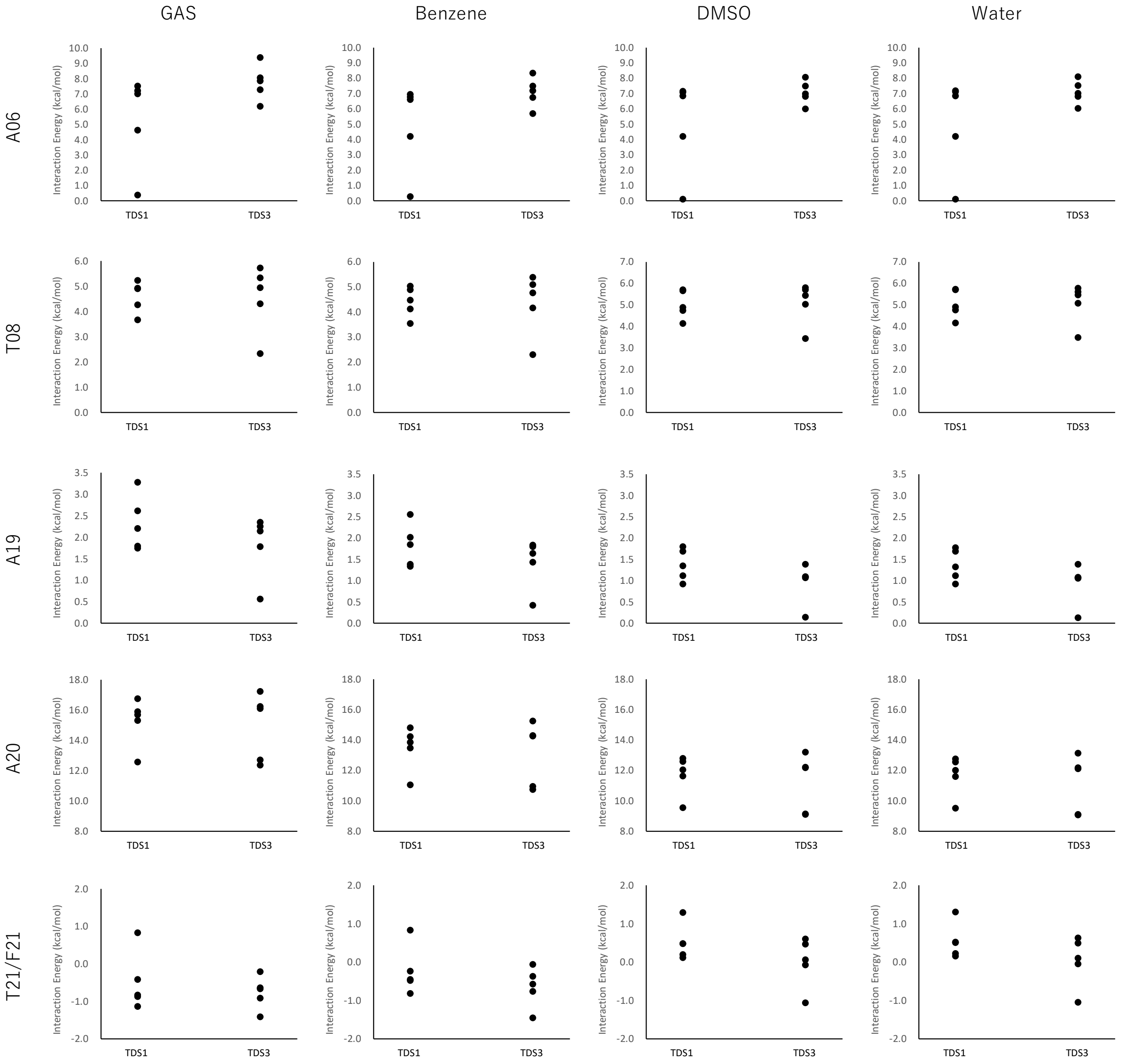

Supplementary Figure 5. Dot plots of interbase interaction energies in some solvent environments (GAS, Benzene, DMSO, and Water phases) between 7th-thymidine (T07) / 7th-trifluorothymidine (F07) and surrounding bases with MP2 levels. The 6-31G* basis set was used. In this table, the positive and negative values correspond to attractive and repulsive interactions, respectively.

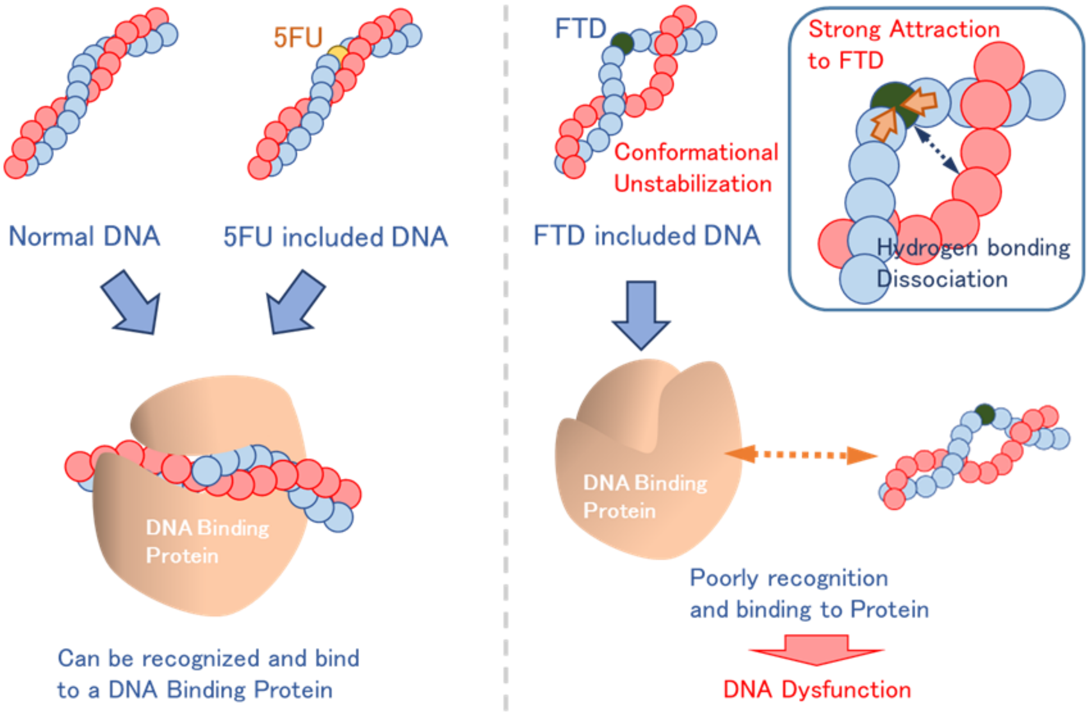

Supplementary Figure 6. Schematic diagram of DNA dysfunction induced by incorporation of FTD into DNA.

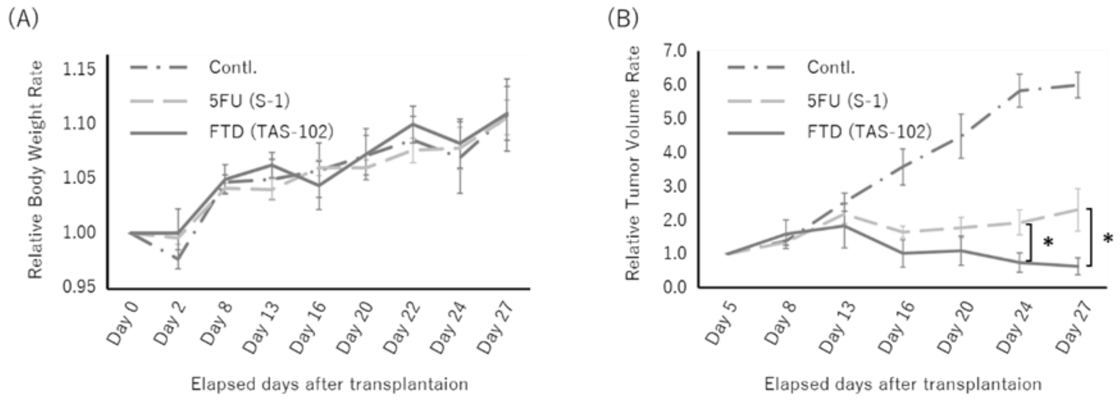

Supplementary Figure 6. The results of the antitumor drugs administration test with the subcutaneous implantation model. The temporal changes of relative body weight rate (A) and relative tumor volume rate (B).

Supplementary Table

Supplementary Table 1. Interbase interaction energies (A) between 7th-thymidine (T07) and surrounding bases in TDS1, and (B) between 7th-trifluorothymidine (F07) and surrounding bases in TDS3 with HF and MP2 levels. The 6-31G* basis set was used. In this table, the positive and negative values correspond to attractive and repulsive interactions, respectively.

(＋) positive : Corresponding to Attractive interaction
(－) negative : Corresponding to Repulsive interaction

(A) TDS1

	HF				
T07 vs	Rep. 1	Rep. 2	Rep. 3	Rep. 4	Rep. 5
A06	0.72	0.05	-0.67	0.31	1.19
T08	-6.52	-1.37	-5.04	-2.36	-4.24
A19	0.86	0.73	1.96	1.10	1.89
A20	9.16	10.78	11.13	11.63	8.84
T21	-2.72	-2.27	-1.95	-1.80	-0.89

[kcal/mol]

	MP2				
T07 vs	Rep. 1	Rep. 2	Rep. 3	Rep. 4	Rep. 5
A06	4.63	7.23	7.01	0.37	7.52
T08	4.90	5.23	4.25	3.67	4.92
A19	2.20	1.79	2.61	1.74	3.27
A20	15.30	15.66	15.86	16.74	12.54
T21	-1.15	-0.88	-0.84	0.82	-0.42

[kcal/mol]

(B) TDS3

	HF				
F07 vs	Rep. 1	Rep. 2	Rep. 3	Rep. 4	Rep. 5
A06	-0.16	-0.52	-0.21	1.48	-0.85
T08	-1.82	-1.34	-1.61	-0.34	-3.75
A19	1.75	1.02	1.34	1.18	0.45
A20	11.91	11.20	11.27	7.14	8.62
T21	-1.65	-2.98	-2.72	-3.99	-1.57

[kcal/mol]

	MP2				
F07 vs	Rep. 1	Rep. 2	Rep. 3	Rep. 4	Rep. 5
A06	6.22	7.29	7.86	9.39	8.06
T08	4.95	5.33	4.30	5.73	2.33
A19	2.35	1.78	2.14	2.25	0.56
A20	17.20	16.23	16.08	12.34	12.67
T21	-0.68	-0.92	-0.65	-1.43	-0.22

[kcal/mol]

Supplementary Table 2. Deference of interbase interaction energies in some solvent environments between 7th-thymidine (T07) / 7th-trifluorothymidine (F07) and surrounding bases with MP2 levels. The 6-31G* basis set was used. In this table, the positive and negative values correspond to attractive and repulsive interactions, respectively.

	Vs T07	TDS1			
		GAS	Benzene	DMSO	Water
A06	ST01	4.626	4.213	4.203	4.215
	ST02	7.231	6.802	7.157	7.193
	ST03	7.006	6.629	7.085	7.122
	ST04	0.372	0.291	0.119	0.113
	ST05	7.524	6.970	6.854	6.868

	Vs F07	TDS3			
		GAS	Benzene	DMSO	Water
A06	ST01	6.217	5.719	6.016	6.052
	ST02	7.293	6.743	6.990	7.019
	ST03	7.855	7.183	6.805	6.809
	ST04	9.389	8.350	8.088	8.110
	ST05	8.064	7.492	7.510	7.532

	Vs T07	TDS1			
		GAS	Benzene	DMSO	Water
T08	ST01	4.896	4.473	4.865	4.897
	ST02	5.226	5.030	5.648	5.688
	ST03	4.254	4.109	4.721	4.755
	ST04	3.666	3.536	4.115	4.148
	ST05	4.916	4.889	5.684	5.724

	Vs F07	TDS3			
		GAS	Benzene	DMSO	Water
T08	ST01	4.947	4.753	5.699	5.754
	ST02	5.329	5.080	5.794	5.604
	ST03	4.301	4.159	5.007	5.057
	ST04	5.727	5.368	5.429	5.443
	ST05	2.330	2.296	3.420	3.481

	Vs T07	TDS1			
		GAS	Benzene	DMSO	Water
A19	ST01	2.198	1.838	1.681	1.684
	ST02	1.787	1.380	1.106	1.105
	ST03	2.613	2.018	1.337	1.321
	ST04	1.742	1.327	0.919	0.912
	ST05	3.273	2.553	1.792	1.775

	Vs F07	TDS3			
		GAS	Benzene	DMSO	Water
A19	ST01	2.350	1.793	1.083	1.066
	ST02	1.776	1.426	1.062	1.056
	ST03	2.140	1.632	1.085	1.073
	ST04	2.251	1.829	1.383	1.375
	ST05	0.563	0.414	0.136	0.128

	Vs T07	TDS1			
		GAS	Benzene	DMSO	Water
A20	ST01	15.304	13.463	11.597	11.554
	ST02	15.657	13.835	12.026	11.985
	ST03	15.861	14.192	12.550	12.513
	ST04	16.745	14.779	12.765	12.717
	ST05	12.543	11.027	9.512	9.477

	Vs F07	TDS3			
		GAS	Benzene	DMSO	Water
A20	ST01	17.202	15.230	13.172	13.123
	ST02	16.234	14.265	12.142	12.090
	ST03	16.083	14.220	12.192	12.142
	ST04	12.342	10.713	9.072	9.035
	ST05	12.673	10.921	9.108	9.066

	Vs T07	TDS1			
		GAS	Benzene	DMSO	Water
T21/F21	ST01	-1.154	-0.824	0.106	0.140
	ST02	-0.879	-0.483	0.468	0.501
	ST03	-0.836	-0.469	0.473	0.507
	ST04	0.822	0.817	1.281	1.303
	ST05	-0.424	-0.248	0.194	0.209

	Vs F07	TDS3			
		GAS	Benzene	DMSO	Water
T21/F21	ST01	-0.676	-0.388	0.458	0.491
	ST02	-0.919	-0.778	-0.084	-0.057
	ST03	-0.648	-0.584	0.058	0.086
	ST04	-1.426	-1.467	-1.074	-1.056
	ST05	-0.224	-0.074	0.595	0.622

Supplementary Table 3. Relative body weight and tumor volume rate for the antitumor drug administration tests with subcutaneous implantation model.

Group		Body weight [g]								
		Day 0	Day 2	Day 8	Day 13	Day 16	Day 20	Day 22	Day 24	Day 27
Contl.	Ave.	1.000	0.977	1.046	1.049	1.057	1.070	1.084	1.068	1.107
	S.D.	0.000	0.015	0.010	0.031	0.043	0.031	0.036	0.055	0.056
	S.E.	0.000	0.009	0.006	0.018	0.025	0.018	0.021	0.032	0.032
GEM	Ave.	1.000	0.961	1.034	1.045	1.028	1.093	1.067	1.097	1.123
	S.D.	0.000	0.011	0.004	0.036	0.060	0.050	0.030	0.025	0.043
	S.E.	0.000	0.006	0.002	0.021	0.035	0.029	0.017	0.014	0.025
5FU (S-1)	Ave.	1.000	0.996	1.041	1.039	1.059	1.059	1.075	1.077	1.104
	S.D.	0.000	0.010	0.007	0.016	0.012	0.012	0.019	0.033	0.027
	S.E.	0.000	0.006	0.004	0.010	0.007	0.007	0.011	0.019	0.016
FTD (TAS-102)	Ave.	1.000	1.000	1.049	1.061	1.043	1.071	1.098	1.081	1.108
	S.D.	0.000	0.038	0.023	0.020	0.038	0.040	0.029	0.038	0.042
	S.E.	0.000	0.022	0.013	0.012	0.022	0.023	0.017	0.022	0.024

Group		Tumor volume [mm^3]						
		Day 5	Day 8	Day 13	Day 16	Day 20	Day 24	Day 27
Contl.	Ave.	1.000	1.395	2.532	3.580	4.488	5.840	6.009
	S.D.	0.000	0.251	0.462	0.927	1.122	0.841	0.658
	S.E.	0.000	0.145	0.267	0.535	0.648	0.485	0.380
GEM	Ave.	1.000	1.153	2.102	1.857	1.724	1.676	1.476
	S.D.	0.000	0.168	1.802	1.859	1.969	2.002	1.775
	S.E.	0.000	0.097	1.040	1.073	1.137	1.156	1.025
5FU (S-1)	Ave.	1.000	1.355	2.174	1.642	1.769	1.926	2.304
	S.D.	0.000	0.114	0.495	0.307	0.532	0.639	1.079
	S.E.	0.000	0.066	0.286	0.177	0.307	0.369	0.623
FTD (TAS-102)	Ave.	1.000	1.584	1.837	1.012	1.098	0.734	0.631
	S.D.	0.000	0.728	1.160	0.706	0.747	0.498	0.443
	S.E.	0.000	0.421	0.670	0.408	0.431	0.287	0.256

Supplementary Movie's Legends

Supplementary Movie 1. A part of TDS1 Trajectory in molecular dynamics simulation.

Supplementary Movie 2. A part of TDS2 Trajectory in molecular dynamics simulation.

Supplementary Movie 3. A part of TDS3 Trajectory in molecular dynamics simulation.

Supplementary Movie 4. A part of adenine-FTD oscillation in TDS3's MD trajectory.
